# A strategy for reducing maternal and newborn deaths by 2015 and beyond

**DOI:** 10.1186/1471-2393-13-216

**Published:** 2013-11-22

**Authors:** Gary L Darmstadt, Tanya Marchant, Mariam Claeson, Win Brown, Saul Morris, France Donnay, Mary Taylor, Rebecca Ferguson, Shirine Voller, Katherine C Teela, Krystyna Makowiecka, Zelee Hill, Lindsay Mangham-Jefferies, Bilal Avan, Neil Spicer, Cyril Engmann, Nana Twum-Danso, Kate Somers, Dan Kraushaar, Joanna Schellenberg

**Affiliations:** 1 Global Development Division, Bill & Melinda Gates Foundation, PO Box 23350, Seattle, WA 98102, USA; 2 IDEAS project, Faculty of Infectious and Tropical Disease, London School of Hygiene and Tropical Medicine, Keppel St, London, UK; 3 IDEAS project, Faculty of Epidemiology and Population Health, London School of Hygiene and Tropical Medicine, Keppel St, London, UK; 4 Centre for International Health and Development, Institute of Child Health, University College London, London, UK; 5 IDEAS project, Faculty of Public Health and Policy, London School of Hygiene and Tropical Medicine, Keppel St, London, UK; 6 Management Sciences for Health, Boston, MA, USA

## Abstract

**Background:**

Achievement of Millennium Development Goal (MDG) 4 for child survival requires acceleration of gains in newborn survival, and current trends in improving maternal health will also fall short of reaching MDG 5 without more strategic actions. We present a Maternal Newborn and Child Health (MNCH) strategy for accelerating progress on MDGs 4 and 5, sustaining the gains beyond 2015, and further bringing down maternal and child mortality by two thirds by 2030.

**Discussion:**

The strategy takes into account current trends in coverage and cause-specific mortality, builds on lessons learned about what works in large-scale implementation programs, and charts a course to reach those who do not yet access services. A central hypothesis of this strategy is that enhancing interactions between frontline workers and mothers and families is critical for increasing the effective coverage of life-saving interventions. We describe a framework for measuring and evaluating progress which enables continuous course correction and improvement in program performance and impact.

**Summary:**

Evidence for the hypothesis and impact of this strategy is being gathered and will be synthesized and disseminated in order to advance global learning and to maximise the potential to improve maternal and neonatal survival.

## Background

Knowledge of what is needed to improve maternal and newborn survival in low-income settings has advanced substantially since the Millennium Development Goals (MDGs) were set in 2000. Evidence to date suggests, however, that only a few of the high-mortality countries will reduce child mortality by two-thirds between 1990 and 2015 (MDG4) and reduce maternal mortality by three-quarters during the same time period (MDG5) [1].

While maternal survival has improved substantially worldwide since 1990, with a 1.9% annual decline in mortality between 1990 and 2011, deaths continue to be concentrated in sub-Saharan African and South Asian countries where the lifetime risk of a woman dying from pregnancy-related causes is about 100 times higher than that of a woman in a high income country [2]. During the same period, child survival (to 5 years of age) also improved markedly, although progress has varied dramatically across income groups and geographies [3]. Newborn survival (to 28 days after childbirth) has improved more slowly in all regions of the world, and globally in 2012 44% of all under-five deaths occurred during the neonatal period [4], up from 37% in 1990. Each year, an estimated 6.6 million children under five years of age die, which includes an estimated 2.9 million newborn infants [3]. Additionally, an estimated 2.6 million babies are stillborn annually [5], primarily in settings where vital registration and cause-of-death statistics are often lacking and maternal and neonatal mortality remain high.

Maternal death can have catastrophic consequence for the whole family [6] and child deaths are linked to maternal health via perinatal causes (stillbirths and early neonatal deaths) [7,8], and via suboptimal care and nutrition in pregnancy and early infancy [9]. Better maternal health and nutrition can improve intrauterine growth and reduce the chance of a low birth weight baby; and subsequently, reduce the risk of stunting, infectious diseases, neurodevelopmental impairments and death [10-13]. Newborns and mothers are both at the highest risk of dying around childbirth: about one-third of neonatal deaths occur in the first 24 hours after birth [14], and the risk of maternal death is highest within 48 hours of delivery, not accounting for the estimated 13% of maternal deaths related to abortion [15,16].

To address this unfinished agenda, we present a strategy for accelerating progress on MDG 4 and 5 leading up to the 2015 target date, sustaining the gains beyond 2015, and further bringing down maternal, neonatal and child mortality by two thirds by 2030. This provides a frame work for measuring and evaluating progress towards these ambitious but feasible goals. The Maternal Newborn and Child Health (MNCH) strategy takes into account current trends in coverage and cause-specific mortality, builds on implementation lessons learned of what works to date, and will help to reach out to those who do not yet access services.

## Discussion

### Towards an MNCH strategy for scale up

A limited number of conditions account for the majority of maternal deaths [6] (haemorrhage, hypertensive disorders, sepsis/infections, and obstructed labour), which also contribute to the highest burden of newborn conditions [17] (preterm birth, severe infections (sepsis, meningitis), and intrapartum-related complications also known as “birth asphyxia”). Life-saving interventions that can be delivered at community level up to first referral level of the health system are well understood but coverage of these interventions remains unacceptably low [18,19]. Global reviews of evidence on the impact and coverage of interventions have been compiled [20,21], informing this strategy for scale up which aims to complement and fill gaps in global action for women and newborns, and proposes a pathway towards sustained health impact.

**Figure 1:**
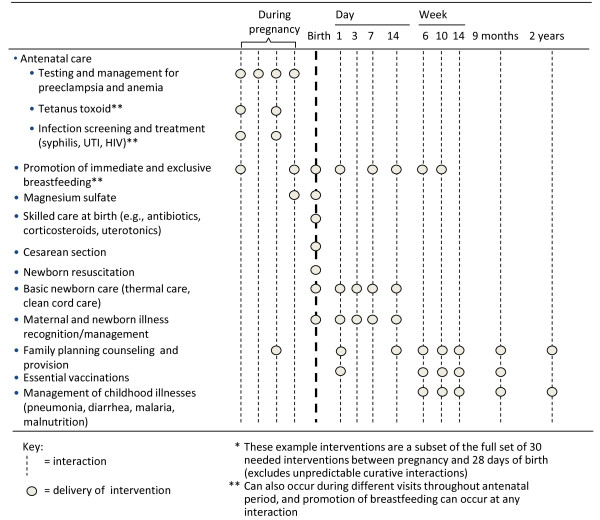
Examples of interventions that can be delivered through interactions between families and frontline workers to reduce neonatal and maternal mortality.

Trained frontline workers, including qualified or unqualified medical practitioners, private drug sellers, community health workers (CHWs), traditional birth attendants, and trained midwives and other skilled birth attendants (e.g., nurses) together provide a critical link to address the problem of low coverage of interventions [21]. In linking cadres of frontline workers who are primarily community-based with those who work in primary health facilities, a larger number of families can be supported through combined counselling, health education and negotiation at home, pregnancy care, skilled care at birth, and postnatal healthcare in communities and primary health facilities (Figure 1) [22]. By connecting communities with the health system [23], for example by mobilizing and empowering families to seek health care with birth preparedness planning or through communication and referral systems, life-saving interventions can be brought closer to those who need them [24], particularly the poorest, who continue to experience the highest burden of mortality [25]. For example in Bangladesh, CHWs demonstrated effective prevention and management of serious neonatal illnesses using interventions such as clean cord care, thermal care, and sepsis management in the home, leading to a 34% reduction in neonatal mortality [26].

Building on this evidence base, this MNCH strategy focuses on behaviour change both at home and in primary health facilities where childbirth services are available, by families and health providers, and strengthens the interconnections between maternal and newborn health, and between frontline workers and families, ensuring that they are well connected to accessible, good quality, clinical services. Demand for facility births is increasing [20], resulting in changes in the rates of facility births globally. To respond to this demand and to other enhanced care seeking practices, attention to the quality of services provided to pregnant women, their newborn babies and to sick children at first level facilities is critical for achieving impact on maternal, newborn and child deaths [27].

### The theory of change

**Figure 2:**
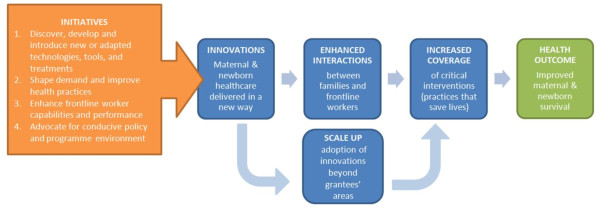
Theory of change.

The MNCH strategy is based on a theory of change (Figure 2) which charts a pathway towards impact on maternal and neonatal survival. Both supply and demand are critical, as is a policy environment which supports program effectiveness. The theory includes initiatives which work across the continuum from discovery and development of tools and technologies to the implementation of delivery strategies that lead to high, equitable, and cost-effective coverage of key interventions.

Enhancing interactions between families and frontline workers is at the heart of this theory of change. For more life-saving interventions to be adopted and spread, more families need to have frequent contacts with skilled and motivated frontline workers who provide good quality care (both from the technical content and user perspectives) in an equitable and pro-poor way (see Additional file 1: Web annex 1 for a list of indicators for enhanced interactions). Demand and supply side innovations which aim to enhance interactions and which are designed for scale-up are currently being tested in the states of Bihar and Uttar Pradesh in India, Ethiopia, and northern Nigeria (see Additional file 1: Web annex 2 for a list of current investments in this strategy by the Bill and Melinda Gates Foundation (BMGF). These geographies account for approximately six percent of the global population and ten percent of global births, but as much as 16 percent of global maternal and neonatal mortality [19]. The BMGF is also investing in the achievement of impact at scale in 8 countries of Mesoamerica, through performance-based financing.

Each country has many potential large-scale delivery vehicles for scale-up and spread—through government programs, private sector, and networks of individuals, communities and organizations. In addition, other non-health sector determinants of maternal and neonatal health are important for accelerated progress, including secondary education of girls, the nutritional status of adolescent girls and access to, and use of clean water and sanitation facilities. Overcoming gender barriers within households and at the community level to accessing health and other services are essential to reach the 2015 MDG targets, and to accelerate progress among the poorest communities that are lagging behind -- beyond 2015.

### A framework for measurement, learning and evaluation of the MNCH strategy

Measurement can generate evidence about what works and what doesn’t work or has unintended consequences. It is an important component in any program strategy in order to enable course correction for program improvement, and to maximize the benefits of local and global action. The measurement framework for this MNCH strategy, developed by the BMGF MNCH strategy team and the IDEAS project (Informed Decisions for Actions in Maternal and Newborn Health, http://ideas.lshtm.ac.uk/), aims to monitor implementation progress and to find out whether the policies and actions proposed in the theory of change are effective in achieving program impact, in different geographic locations.

Specifically, the measurement, learning and evaluation answer the following questions:

(1) What community-based maternal and newborn health innovations are being implemented in Ethiopia, northern Nigeria and northern India, and through what pathways and processes are they expected to increase “effective coverage” of key interventions (effective coverage being the fraction of the potential health gain of an intervention that is being delivered to a population)?

(2) To what degree do these innovations enhance interactions between families and frontline workers, and increase intervention coverage, in program areas? Are they cost-effective? Through what mechanisms do enhanced interactions affect coverage of key interventions?

(3) What helps and what hinders scale-up of innovations, both within and beyond project areas, and how can scale-up be catalysed and leveraged for impact beyond program bounds?

(4) Where innovations have been implemented on a large scale beyond program areas (through government programs, markets and networks), what is the effect on coverage of key interventions and how does this depend on implementation strength? What survival impact can be expected?

The evaluations are multi-disciplinary and include both qualitative and quantitative approaches. For the latter, IDEAS , BMGF and program implementers and partners are using quasi-experimental plausibility designs [28], with emphasis on data quality and use of monitoring as well as evaluation results. Taken as a whole, the approach uses data collected in support of all components of the theory of change in order to track implementation progress by foundation grantees and by other partners when innovations spread through catalytic effects. Data is used to make evidence-based decisions about program improvement for enhanced efficiency, effectiveness and equity, and to generate evidence of impact and learning for future investments.

### Implications for research and policy

In developing the measurement framework, several key principles for effective monitoring and evaluation were applied. First, it is important that efforts have country ownership and are aligned with country models and measurement efforts. Involving in-country researchers and policymakers during evaluation design is of central importance. This MNCH strategy and measurement framework aims to complement existing structures at the country level, and not lead to parallel data collection systems or processes. Primary data is collected only to fill gaps. Since the measurement is applied to the “real world” of field implementation, evaluation design is often constrained by lack of valid comparison areas: defining the scale and context in which innovations are implemented is therefore an essential component.

Results of measurement efforts must be fed back to program implementers in a timely way so that the maximum possible use of data is made for course correction. Results must also be shared widely, particularly with decision and policy makers who can make policy and program changes to improve health services for women and children. Beyond the country-level, the new evidence that will be generated from these evaluation activities is anticipated to show the extent to which large-scale delivery strategies maximize frontline worker potential to increase coverage of life-saving interventions; this evidence will be relevant for others striving to improve the survival of mothers and newborns.

### Contribution to global efforts

Since the launch of the MNCH strategy by the BMGF, adjustments are being made to reflect changes in the global landscape and lessons learned through implementation. For example, as mortality rates decline and the cause structure of mortality changes [29], with preterm birth now being the second-leading cause of under-five deaths, increased emphasis is given by global partners to the prevention and management of prematurity. As frontline health worker programs are rolled out, for example in India and Ethiopia, the focus on quality and equity is increasing, in addition to the number of interactions with families. Similarly, as demand is generated for facility services and births increasingly take place in health facilities, greater emphasis is placed on quality improvement activities for care, as well as more comprehensive care at childbirth [30]. Finally, the importance and role of partners working together towards common outcomes will be critical for achieving the post 2015 MDGs. We estimate based on solid trends analysis by the Child Health Epidemiology Reference Group, USAID and UNICEF that two thirds of maternal and childhood deaths could be averted by 2030 and that we collectively should be held accountable for achieving those ambitious but realistic goals [31].

## Summary

In conclusion, this MNCH strategy provides an effective framework for priorities and actions, measurement and evaluation, and can guide decisions about the scope of investments along the pathway towards impact. It is based on a theory of change that is oriented towards addressing the highest risks of dying for mothers and newborns. The strategy proposes innovative potential solutions (Additional file 1: Web annex 2) to mitigate those risks, with a focus on enhancing interactions between frontline workers and mothers and families as a critical lever in increasing the effective coverage of life-saving interventions. When new innovations are introduced to health services, measurement must be incorporated in order to monitor progress along the way [32]. Strategy and measurement are intertwined: identifying the link between action and impact can validate strategies, identify the most effective innovations to take forward, and inform course correction in strategy, investments and implementation. To take forward innovative local solutions to achieve impact at scale, strategic and catalytic partnerships are essential, and increasingly such partnerships are formed with governments providing the leadership and with other development partners engaged, for example, in the states of Bihar and UP in India, and in Nigeria, Malawi and Ethiopia. This strategy, strengthened by its measurement framework, should contribute to the overall global efforts to improve maternal and newborn survival, reducing deaths by two thirds by 2030. Evidence for this hypothesis is being gathered and will be synthesized and disseminated, in order to advance global learning and to maximise the potential to improve maternal and neonatal survival.

## Competing interests

The authors declare that they have no competing interests.

## Authors’ contributions

JS, GLD, TM and MC conceived of the concept for the article. TM and GLD wrote the first draft. All authors contributed to the intellectual content and writing process of the paper. GLD is the overall guarantor. All authors read and approved the final manuscript.

## Pre-publication history

The pre-publication history for this paper can be accessed here:

http://www.biomedcentral.com/1471-2393/13/216/prepub

## Supplementary Material

Additional file 1 **Web Annex 1.** Indicators for enhanced interactions (more and better) between families and frontline workers across the continuum of care. **Web annex 2.** Current investments by the Bill & Melinda Gates Foundation in innovations to enhance interactions between families and frontline workers (initiatives 2 and 3) in Ethiopia, Northern Nigeria, and Uttar Pradesh, India. Click here for file
