# Supplementary material for: A strategy for reducing maternal and newborn deaths by 2015 and beyond
Source: BMC Pregnancy Childbirth. 2013 Nov 22;13:216. doi: 10.1186/1471-2393-13-216 (PMC3866510; doi:10.1186/1471-2393-13-216)
Supplement: Additional file 1 — Web Annex 1. Indicators for enhanced interactions (more and better) between families and frontline workers across the continuum of care. Web annex 2. Current investments by the Bill & Melinda Gates Foundation in innovations to enhance interactions between families and frontline workers (initiatives 2 and 3) in Ethiopia, Northern Nigeria, and Uttar Pradesh, India. [file 1471-2393-13-216-S1.docx]

**Web Annex 1. Indicators for enhanced interactions (more and better) between families and frontline workers across the continuum of care**

| **Stage** | **Indicator** |
| --- | --- |
| **More pregnancy care interactions** | |
| 1 | Mean number of pregnancy interactions per woman^1^ |
| 2 | Percent of women who had any pregnancy care^1^ |
| 3 | Percent of women who attended a health facility at least once for antenatal care^1^ |
| 4 | Percent of women who had a least one antenatal care interaction with a skilled provider^1^ |
| 5 | Percent of women who had a least four pregnancy care interactions (with any provider)^1^ |
|  |  |
| **Better pregnancy care interactions** | |
| 1 | Percent of women who had (unprompted) knowledge of at least one danger sign in pregnancy (valid responses included: severe headaches, blurred vision, reduced foetal movement, high blood pressure, convulsions, excessive bleeding, severe abdominal pain, high fever, anaemia)^1^ |
| 2 | Percent of women who reported having received good quality pregnancy care (components included weight, height and blood pressure measured, urine and blood tested; counselled for breastfeeding, danger signs, birth preparedness)^1^ |
| 3 | Percent of women who reported having made any preparations for delivery (components included finances, transport, food, birth attendant, identified facility)^1^ |
| 4 | Median gestation at first pregnancy care interaction^1^ |
| 5 | Percent of primary level facilities with all essential commodities needed to deliver antenatal care^2^ |
| 6 | Percent of frontline workers who have knowledge of the components of focussed antenatal care^2^ |
|  |  |
| **More intra-partum interactions** | |
| 1 | Percent of women who delivered in a health facility^1^ |
| 2 | Percent of women who were attended by a skilled attendant during last birth^1^ |
| 3 | Percent of women who were advised to seek extra care during intra-partum period who did seek extra care^1^ |
| 4 | Percent of births delivered by caesarean section^1^ |
|  |  |
| **Better intra-partum interactions** | |
| 1 | Women with (unprompted) knowledge of at least one intra-partum danger sign (valid responses included: vaginal bleeding, foul discharge, high fever, foetus hand or feet present first, foetus in abnormal position, prolonged labour, retained placenta, ruptured uterus, prolapsed cord, cord around newborns neck, convulsions)^1^ |
| 2 | Percent of health facilities with essential commodities needed for intra-partum care^2^ |
| 3 | Percent of frontline workers able to prepare essential items for the last birth they attended (items included: sterile gloves, clean cloths and gauze, sterile cord cutter, cord ligature, disinfectant, uterotonic^2^ |
|  |  |
| **More postpartum interactions** | |
| 1 | Percent of women who had at least one postpartum check within 2 days of birth^1^ |
| 2 | Percent of women who had at least one postpartum check within 7 days of birth^1^ |
|  |  |
| **Better postpartum interactions** | |
| 1 | Percent of women who reported receiving good quality postpartum care (components included breasts checked, bleeding checked, counselled for: danger signs, family planning, nutrition) ^1^ |
|  |  |
| **More postnatal interactions** | |
| 1 | Percent of newborns who had at least one postnatal check within two days of birth^1^ |
| 2 | Percent of newborns who had at least one postnatal check within seven days of birth^1^ |
| 3 | Percent of mothers whose newborn had at least one danger sign in the first 28 days of life who sought care for that danger sign outside the home^1^ |
|  |  |
| **Better postnatal interactions** | |
| 1 | Percent of newborns receiving good quality postnatal care (components include newborn weighed, cord checked, mother counselled on breastfeeding, thermal care, and newborn danger signs) ^1^ |
| 2 | Percent of mothers of newborns who had (unprompted) knowledge of at least one newborn danger sign (valid responses include fever, difficulty feeding or suckling, difficult breathing, diarrhoea, convulsions, vomiting, jaundice, lethargy, unconsciousness)^1^ |
| 3 | Percent of health facilities with the essential commodities needed to deliver postnatal care^2^ |
| 4 | Percent of frontline workers with knowledge of appropriate care for the low birthweight baby (including thermal care, supporting breastfeeding, monitoring newborn, and ensure infection prevention)^2^ |

^1^ Measured through household surveys, interviewing women with a live birth in the 12 months preceding survey

^2^ Measured through health facility and frontline worker surveys, recording availability of supplies on the day of survey

**Web Annex 2. Current investments by the Bill and Melinda Gates Foundation in innovations to enhance interactions between families and frontline workers (initiatives 2 and 3) in Ethiopia, Northern Nigeria, and Uttar Pradesh, India**

The current investments in innovations have been categorized as affecting demand or supply as follows:

| **Type of Innovation** | **COUNTRY** | | **List of Projects and Innovations.** Where an innovation includes both demand and supply it is described under both. |
| --- | --- | --- | --- |
| **DEMAND** | **Ethiopia** | **Last 10 kilometres (L10K)^1^:**   1. Targeted **Family Conversations** conducted by health extension workers and the health development army with key family members to improve maternal and newborn care-seeking, and enhance the number and timing of their interactions. 2. **Participatory Community Quality Improvement** is a framework for continuous quality improvement through a cyclical process to identify barriers to quality of services, develop an action plan to address barriers, implement the action plan and assess quality improvement solutions. 3. The **Community Solutions Fund** makes small funds (up to $1500) available to kebele-level institutions, enabling them to address barriers to quality Maternal Newborn and Child Health services and to propose and implement innovative strategies to address them.   **The Maternal and Newborn Health in Ethiopia Partnership (ManHEP)^2^:**   1. **Behaviour Change and Communication Strategy** develops and uses behaviour change communication and marketing strategies to change behaviour and thereby enhance safe Maternal and Newborn Health-related behaviour in the community. 2. **Family meetings/ Community gatherings** enhance Maternal and Newborn Health knowledge in the community, advise families and community members on Maternal and Newborn Health practices and prompt pregnant women to register or seek care. 3. **Performance monitoring** Woreda and health centre staff trained as coaches for frontline workers and the frontline worker teams for quality improvement activities;   **COMBINE^3^**:   1. **Enhance Maternal and Newborn Health profile in the community** - a community-based innovation to enhance knowledge and raise profile of Maternal and Newborn Health among community members.      1. **Male Volunteers for Maternal and Newborn Health** - an innovation to support male community volunteers to promote awareness of newborn health and support referral where necessary, using existing community structures. | |
|  | **Nigeria** | **Society for Family Health**^4^:   1. The **Forum of Mothers-in-Law** is established by Traditional Birth Attendants (TBA) and Federation of Muslim Worker Association of Nigeria (FOMWAN) volunteers to promote messages on safe pregnancy, delivery and postnatal care for the mother and newborn and to enhance interactions between mothers-in-law and pregnant / recently-delivered women. 2. **Advocacy training for men** is an innovation to promote community-based advocacy for Maternal and Newborn Health whereby Federation of Muslim Worker Association of Nigeria volunteers promote peer education in Maternal and Newborn Health among male community members using specifically-designed materials. 3. The **Mass Media Event** for behaviour change comprises a range of approaches to spreading Maternal and Newborn Health messages, e.g. radio spots and bill-boards to promote the Call Centre and publicise contact telephone numbers among community members. | |
|  | **India** | **Sure Start^5^:**   1. Innovation to **Revitalise Village Health & Sanitation Committees (VHSC)** functions by non-governmental organisations working with existing village health and sanitation committees to make and help execute a village health plan for Maternal and Newborn Health. 2. **"The First Hour”** is a communication campaign to popularise key Maternal and Newborn Health messages through games, advertisements, etc to bring about change in behaviour in key post-delivery practices in the community. 3. Through the **Mothers Groups** innovation, Sure Start non-governmental organisation partners mentor and equip Accredited Social Health Activists (ASHAs) to conduct effective Mothers’ Group meetings including themed discussions using the Accredited Social Health Activists toolkit to convey key messages. 4. The **Letter for my Father** is an emotional appeal from the unborn child to the father to take care of its mother during pregnancy, delivery preparation and the delivery. | |
| **SUPPLY** | **Ethiopia** | **Last 10 Kilometres (L10K)^1^:**   1. **Health Extension Worker (HEW) Training and Supervision** complements the standard Health Extension Worker training programme, with emphasis on strengthening communication and negotiation with the community and refreshers in key technical areas, particularly practice in pregnancy and postpartum care of the mother and newborn and identification of intra-partum danger signs, and to train Health Extension Workers to enhance the existing training package for the Health Development Army. 2. **Health Development Army (HDA) training** provided by Health Extension Workers on effective interactions with households to enhance compliance with recommended Maternal and Newborn Health care. 3. **Community Anchors** support the health development army at community meetings and offer continued support and encouragement to the Health Development Army by working through existing community institutions such as women’s organisations, youth associations, churches and mosques. 4. **Non-Financial Incentives** for Community Health Promoters and the Health Development Army for their work with pregnant women, women in labour, new mothers and their families. 5. **Health Extension Worker and Health Development Army Toolkit** comprises materials for use by Health Extension Workers in their interactions with mothers and families and as they train Health Development Army volunteers. 6. **Improved referral linkages** to enhance Health Development Army members’ knowledge of when, why, and how to refer and their capacity to refer, as well as structural mechanisms to facilitate referral by Health Extension Workers to the Health Centre and by the Health Centre to the hospital. 7. Under the **Community-Based Data for Decision-Making** innovation, Health Extension Workers and Health Development Army map households in their catchment areas and produce information on Maternal and Newborn Health which is reviewed routinely and used to identify gaps in provision and as a basis for prioritising effort in collective action. 8. The **Family Folder** is a standardised household-based medical record for use by Health Extension Workers to increase completeness in case management and to enhance data management. 9. **Participatory Community Quality Improvement** is a framework for continuous quality improvement through a cyclical process to identify barriers to quality of services, develop an action plan to address barriers, implement the action plan and assess quality improvement solutions.   **The Maternal and Newborn Health in Ethiopia Partnership (ManHEP)^2^:**   1. **Maternal and Newborn Health care training package** to develop skills of frontline workers and community members to provide targeted Maternal and Newborn Health service in pregnancy, around the time of birth and up to 48 hours thereafter. 2. **Frontline worker team development,** an innovation to develop effective Maternal and Newborn Health teams comprising lay community members and health professionals, which enhance work of frontline workers and the community to provide targeted Maternal and Newborn Health services during pregnancy, delivery and 48 hours thereafter; 3. **Performance monitoring** Woreda and health centre staff trained as coaches for frontline workers and the Frontline worker teams for quality improvement activities.     **COMBINE^3^**:   1. **Community Health Promoter Training** to enhance identification and registration of pregnant women, counselling on antenatal care, safe intra-partum practice, postnatal care of the mother and newborn care. 2. **Health Extension Worker Training** complements standard training to enhance their practice in pregnancy and postpartum care of the mother and newborn and identification of intra-partum danger signs, and to train HEWs to enhance existing training package for the Health Development Army. 3. **Community-based administration of antibiotics** to train and equip HEWs to identify newborns with danger signs, and to provide appropriate and timely extended-dose gentamicin regimen for infections at home or at health posts. 4. **Health Extension Worker/ Health Development Army linkages** - an innovation to enhance communication between the Health Extension Workers and the Health Development Army. | |
|  | **Nigeria** | **Society for Family Health^4^**:   1. **Prepare, train and deploy and support community-based Frontline workers** in rural areas (Traditional Birth Attendants (TBA) and Pastoral Resolve NGO (PARE)) and in urban areas (Federation Of Muslim Worker Association of Nigeria (FOMWAN)) to promote safe pregnancy, intra-partum and postpartum care of the mother and newborn. 2. **Financial Incentives for Frontline worker** comprise a system of financial incentives for Frontline workers who accompany women to a facility for delivery care or who refer a woman or newborn to a facility in response to danger signs and financial incentives for local government areas in recognition of achievement in facility deliveries and facility-based management of referrals. 3. The **Front Line Worker toolkit** is distributed to Frontline workers for use in interactions with women, newborns and families, and comprises a range of job-aids including a flipchart of Maternal and Newborn Health information, practical equipment for home visits and, for less literate volunteers, an innovative approach to logging Maternal and Newborn Health activities. 4. **Call Centre** offering a free telephone helpline to community members, offering information and advice on health behaviour and care-seeking including access to community-based providers, approved facilities and to the Emergency Transport Scheme. 5. The **Emergency Transport Scheme** runs through the National Union of Road Transport Workers to offer low-cost or free transport for facility delivery and in response to an obstetric or newborn emergency. 6. **Upgrade Primary Care Facilities** in collaboration with Saving Newborn Lives (Nigeria), provide technical support (training and equipment) to enhance Maternal and Newborn Health provision in Primary Care Centres, e.g. management of postpartum haemorrhage, newborn asphyxia, clean delivery, Kangaroo Mother Care. 7. **Mapping of service users and provision** enhances existing government mapping of facilities and adds information on quality of care, location of trained Frontline workers, telecom coverage, and pastoralist migration routes, made available to Ministry of Health, Frontline workers and Call Centre staff. 8. **Links with pastoralist / remote or hard to reach communities** is an innovation to identify and contact pastoralist / remote or hard-to reach communities and their gate-keepers to facilitate routine and emergency service provision. 9. **Access to Clean Delivery Kits** is a social marketing innovation to make Clean Delivery Kits available at reduced cost through commercial vendors (Proprietary Patent Medicine Vendors), Traditional Birth Attendants and Federation of Muslim Worker Association of Nigerian volunteers. | |
|  | **India** | **Sure Start^5^:**   1. **Accredited Social Health Activist (ASHA) Mentoring and Supportive Supervision** is an innovation offering ASHAs supportive supervision, using materials in the ASHA toolkit, relating to Maternal and Newborn Health knowledge, skills and working practice. 2. Through the innovation **Recognition of good performance of ASHAs and Village Health & Sanitation Committee (VHSC),** well-performing Accredited Social health Activists and Village Health & Sanitation Committees are given recognition by the community at an open meeting. 3. The **Accredited Social Health Activist (ASHA) Toolkit** comprises information materials for use at home visits and village events to counsel women and their families on the prioritized behaviours. 4. Innovation to **Revitalise Village Health & Sanitation Committees** functions by non-governmental organisations working with existing village health and sanitation committees to make and help execute a village health plan for Maternal and Newborn Health. 5. The **Non-Governmental Organisation (NGO) Partnership architecture** is an innovation which establishes a framework of relationships between 55 existing NGOs which implement the Sure Start project, which facilitates mentoring partnerships between NGO-based supervisor and ASHA, the flow of technical information and support, capacity-building of NGOs.   **Manthan^6^:**   1. **Auxiliary Nurse Midwife (ANM) training in Skilled Birth Assistance** to enhance standard Auxiliary Nurse Midwife training in management of normal deliveries and in immediate newborn care. 2. **mSakhi - a mobile phone**-based work-aid to enhance pregnancy and postnatal care, comprising an educational / knowledge programme to support ASHAs in their interactions with families. 3. **mNewbornCare** - a mobile-phone-based work-aid for ASHAs and Auxiliary Nurse Midwives for use at home visits for newborn care, a checklist to assess condition of the newborn, a management tool for Auxiliary Nurse Midwives to monitor ASHA home visits, with reference to Integrated Management of Newborn and Child Illness information (IMNCI). 4. **Emergency Medical Transport Scheme** for transport of pregnant women and newborns to reduce barriers to timely referral reduce and to increase service uptake among the poor and those living in remote areas. 5. **Mother and Child Tracking System** generates an Auxiliary Nurse Midwife work plan for each mother and newborn, captures completeness of care, enhances follow-up and generates reports to be used for planning services at Block level.   **Better Birth^7^:**   1. The **Safe Childbirth Checklist** is a checklist of actions for each woman and baby cared for during institutional birth and implemented with support at facilities offering routine delivery care. The actions represent best practice at each stage of care from admission to discharge from the facility. | |

^1^ The Last 10 Kilometres project is funded via a grant awarded to John Snow Inc. Website: <http://l10k.jsi.com/>

^2^ The Maternal and Newborn Health in Ethiopia Partnership project is funded via a grant awarded to Emory University. Website: <http://www.nursing.emory.edu/manhep/>

^3^ The COMB INE project is funded via a grant awarded to Saving Newborn Lives. Website: http://www.savethechildren.org/site/c.8rKLIXMGIpI4E/b.6234293/k.6211/Saving_Newborn_Lives.htm

^4^ The Society for Family Health project is funded via a grant awarded to The Society for Family Health. Website: <http://www.sfhnigeria.org/>

^5^ The Sure Start project is funded via a grant awarded to PATH. Website: <http://www.path.org/projects/sure-start.php>

^6^ The Manthan project is funded via a grant awarded to IntraHealth. Website: <http://www.intrahealth.org/page/the-planning-for-improving-maternal-and-neonatal-health-in-northern-india-project>

^7^ The Better Birth project if funded via a grant awarded to Harvard School of Public Health. Website: <http://www.hsph.harvard.edu/>
